# The complete chloroplast genome sequence of *Rhaponticum uniflorum*, the first of the genus *Rhaponticum*

**DOI:** 10.1080/23802359.2022.2029600

**Published:** 2022-01-30

**Authors:** Hu Boqin, Qiang Zhou, Liqiang Wang, Mei Jiang, Guohua Gong, Chang Liu, Chengxi Wei

**Affiliations:** a Medicinal Chemistry and Pharmacology Institute, Inner Mongolia Minzu University, Tongliao, P.R. China; b Inner Mongolia Key Laboratory of Mongolian Medicine Pharmacology for Cardio-Cerebral Vascular System, Tongliao, P.R. China; c College of Pharmacy, Heze University, P.R. China; d Institute of Medicinal Plant Development, Chinese Academy of Medical Sciences and Peking Union Medical College, Beijing, P.R. China; e Affiliated Hospital of Inner Mongolia Minzu University, Tongliao, P.R. China

**Keywords:** *Rhaponticum uniflorum*, chloroplast genome, phylogeny

## Abstract

*Rhaponticum uniflorum* is commonly used as a source for traditional medicines with the main effect of clearing heat. Here, we sequenced the complete chloroplast (cp) genome of *R. uniflorum* to develop molecular markers for taxonomic classification and species determination of *R. uniflorum*. It was 152,760 bp in size and has a typical circular structure, including a pair of inverted repeats with 25,205 bp, a large single-copy region with 83,687 bp, and a small single copy region with 18,663 bp. The genome encodes 110 unique genes, including 80 protein-coding, four rRNA and 26 tRNA genes. Phylogenomic analysis shows that *R. uniflorum* is closely related to the *Saussurea*. The study is useful for phylogenetic and population genetic studies of *Rhaponticum* plants.

*Rhaponticum uniflorum* (L.) DC. (*Rhaponticum uniflorum* (L.) DC. 1810) is a perennial herb and belongs to the *Rhaponticum* genus, Asteraceae family. The root, used directly as a traditional medicine, has the effect of clearing heat, detoxifying, relieving swelling and expelling pus (Committee 2020). *Rhaponticum uniflorum* contains several types of compounds including phytoecdysones, steroids, terpenoids, thiophenes, and flavones (Zhu et al. 1991). Pharmacological studies showed its activities of anti-inflammatory, anti-oxidative, immunomodulating, and anti-tumor effects (Jin et al. 2011; Chen et al. 2017). However, there is little research on the genomic resource of *R. uniflorum*, which limited the species identification and resource conservation. In this study, we report the cp-genome sequence of *R. uniflorum*, which is the first report of chloroplast genome in *Rhaponticum*.

A sample was collected in Dafutou village (Geographic coordinates 115°95′19″N, 40°38′42″E), Zhangshanying Town, Yanqing District, Beijing, China and identified as *R. uniflorum* by Professor Jinwen You. A specimen and its DNA were deposited at Institute of Medicinal Plant Development (www.implad.ac.cn/, Chang Liu and cliu6688@yahoo.com) under the voucher number 2019071712. Genomic DNA was extracted from mature fresh leaves through modified CTAB method (Doyle 1987), and purified to construct a DNA library with the insertion size of 500 bp. Paired-end sequencing was performed on the Hiseq 2500 platform (Illumina, San Diego, CA, USA). The raw sequence data were fed into NOVOPlasty (version 2.7.2) (Dierckxsens et al. 2017) to assemble the cp-genome. Then, the annotation was finished by using CpGAVAS2 (Shi et al. 2019).

The chloroplast genome of *R. uniflorum* (accession number MW683229) is 152,760 bp in size with a pair of inverted repeats (IRs) of 25,205 bp separated by a large single-copy (LSC) region of 83,687 bp and a small single-copy (SSC) region of 18,663 bp. The chloroplast genome encoded129 genes, of which 110 are unique genes including 80 protein-coding, four ribosome RNA (rRNA), and 26 transfer RNA (tRNA) genes. Among them, ten protein-coding genes had one intron and two had two introns. Six tRNA genes were found to contain one intron. The GC content of the genome was 51.47%, of which the protein-coding, the rRNA, and the tRNA genes were 70.6, 55.09, and 51.78%, respectively.

To infer the phylogenetic position of *R. uniflorum*, we constructed a maximum-likelihood (ML) tree using the sequences of the whole chloroplast genomes of twenty-nine species in the Asteraceae family, including two species in the *Ambrosia* used as outgroup. In the phylogenetic tree, *R. uniflorum* is the only species in the *Rhaponticum*, and each of the other genera has two species. 44 shared proteins sequences were subjected to PhyloSuite to construct the ML phylogenomic tree (Zhang et al. 2020). Then, the phylogenomic tree was visualized by using MEGA X (Kumar et al. 2018). As shown in Figure 1, R*. uniflorum* is clustered within the group consisting *Saussurea hookeri* and *S. pubifolia*, and the bootstrap score is greater than 50 and less than 70, indicating that *R. uniflorum* is very closely related to *Saussurea*. Except *R. uniflorum*, all two species from the same genus are clustered together, and some species from the same tribe are clustered together. This study will be useful in phylogenetic and population genetic studies of *Rhaponticum* plants.

**Figure 1.**
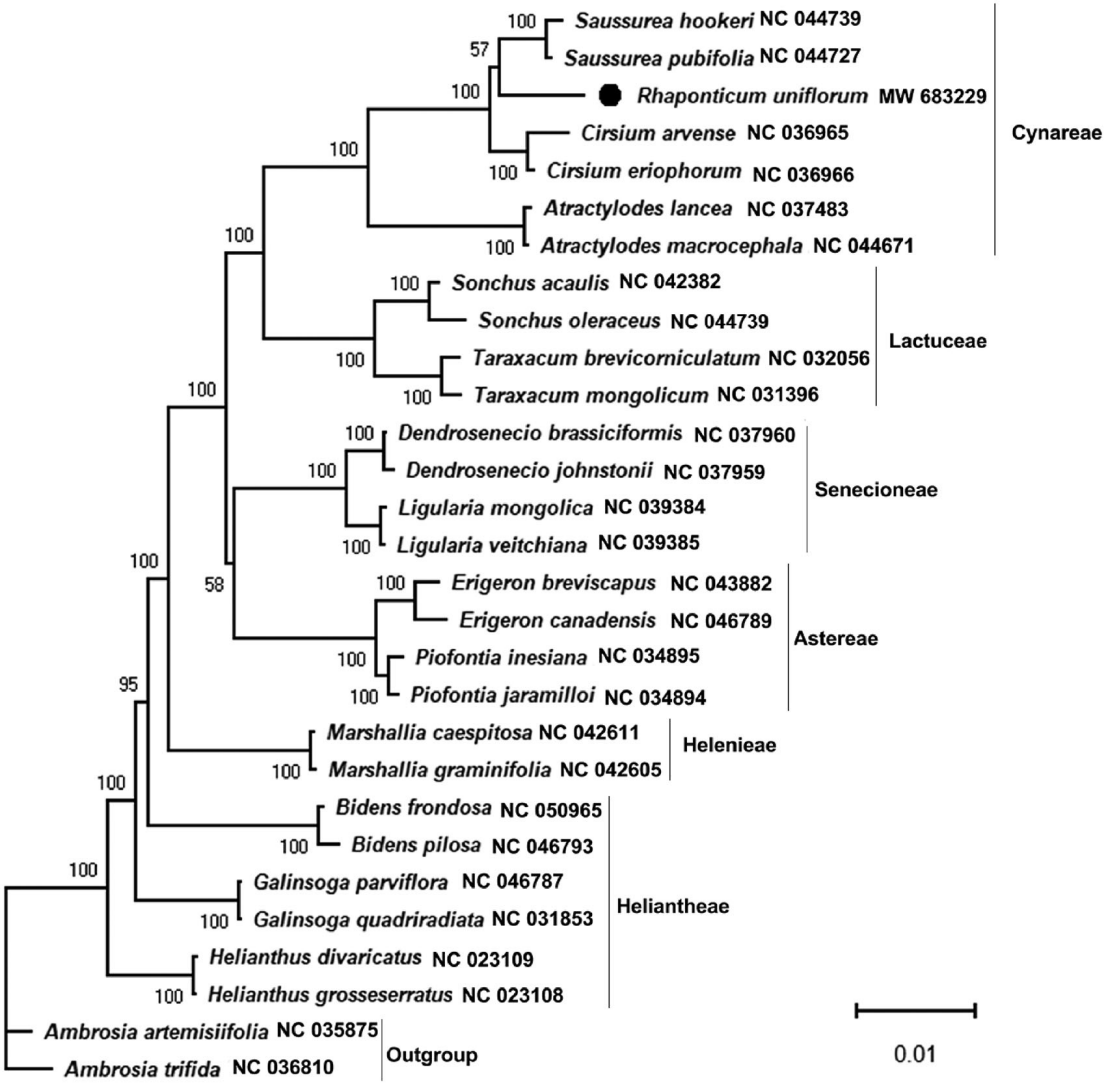
Molecular phylogenetic tree showing the position of *Rhaponticum uniflorum* in the family Asteraceae based on the complete chloroplast genomes among 29 species. The tree was constructed using maximum likelihood (ML) algorithm. Numerical value beside each node shows the bootstrap value obtained from 1000 replications. The branch lengths are scaled with a scale bar. The GenBank accession number for the corresponding sequences is shown to the right of the Latin name.

## Ethics approval and consent to participate

The research, including the collection of plant materials, was carried out in accordance with guidelines provided by the authors’ institutions and national or international regulations.

## Authors’ contributions

The article was designed and conceived by Chang Liu and Chengxi Wei; It was Liqiang Wang, HuBoqin, Qiang Zhou, Mei Jiang and Guohua Gong who got involved in the analysis and interpretation of the data; HuBoqin and Qiang Zhou drafted the article; Chang Liu revised it critically for intellectual content; All authors approved the final version to be published and agreed to be accountable for all aspects of the work.

## Data Availability

The genome sequence data that support the findings of this study are openly available in GenBank of NCBI at (https://www.ncbi.nlm.nih.gov/) under the accession NO. MW683229. The associated BioProject, BioSample, and SRA number are PRJNA722731 and SAMN18790581, and SRR14270266 respectively.
